# Comparison of the Effects of a Genetic, a Mild Encephalitis, and a Psychosocial Causal Explanation of Schizophrenia on Stigmatizing Attitudes – a Pilot Study With a Quasi-Experimental Design

**DOI:** 10.3389/fpsyt.2021.745124

**Published:** 2021-09-20

**Authors:** Sonja Haouchet, Carolin Harder, Sabine Müller

**Affiliations:** Department of Psychiatry and Neurosciences, CCM, Charité - Universitätsmedizin Berlin, Corporate Member of Freie Universität Berlin and Humboldt-Universität zu Berlin, Berlin, Germany

**Keywords:** stigmatization, social distance, schizophrenia, mild encephalitis hypothesis, genetic essentialism, attribution theory, causal beliefs

## Abstract

**Background:** Previous research has shown that the endorsement of biogenetic causal explanations of schizophrenia is associated with stronger stigmatizing attitudes against people with schizophrenia than the endorsement of psychosocial explanations. However, little is known about whether different biogenetic causal explanation beliefs differentially affect stigmatizing attitudes. This is particularly valid for the endorsement of the mild encephalitis hypothesis of schizophrenia.

**Aim:** To examine to what extent different causal explanations of schizophrenia influence the desire for social distance from persons with schizophrenia.

**Methods:** A study with a prospective, quasi-experimental design was carried out with students in Germany (*N* = 333). A case vignette depicting a person with schizophrenia-typical symptoms was presented, and a social distance scale (SDS) was used to measure the stigmatizing attitude against the person described. Participants were randomly assigned to one of three groups receiving different causal explanations of schizophrenia (genetic, mild encephalitis hypothesis, or psychosocial) without treatment information.

**Results:** A one-way ANOVA showed that the mean SDS was lowest in the group with the mild encephalitis hypothesis explanation, followed by the genetic explanation group, and highest in the psychosocial explanation group. However, the differences between the groups were small and not significant. A subanalysis revealed a significant interaction between gender and causal explanation. Women showed a significantly lower desire for social distance than men when receiving the mild encephalitis hypothesis. Neither the study discipline nor the number of semesters of study had significant effects on the mean SDS. The differences between the mean SDS scores for the different items were much bigger than the differences for the different causal explanations. Regardless of the causal explanation, the extent of the desired social distance depends strongly on social proximity.

**Conclusion:** The present study fits into previous research, which has found that biogenetic beliefs were either associated with more social distance or did not yield a statistically significant association. Although we found a small gender-specific effect of the endorsement of the mild encephalitis hypothesis, we do not recommend gender-specific anti-stigmatization campaigns because they might rightly raise suspicions of dishonesty and manipulation. Rather we support recovery-oriented messages focusing on effective treatments.

## Introduction

### Stigmatization

People suffering from psychiatric disorders are severely stigmatized (1–5). The history of stigma goes back to antiquity, when people with mental disorders were perceived as violent, unpredictable, and mad due to being possessed by demons (6, 7). In modern times, persons with mental disorders are still considered dangerous, uncontrollable, or weak (6). People want social distance from persons with mental disorders, mainly from persons with substance use disorders, followed by persons with schizophrenia, depression, and anxiety disorders (8).

According to Link and Phelan, “stigma exists when elements of labeling, stereotyping, separation, status loss and discrimination occur together in a power situation that allows them” (9) (p. 377). Important to note is the dependence of stigma on power, particularly on social, economic, and political power (9). Stigmatization often includes the social circle, particularly the families of stigmatized persons (courtesy stigma) (10). The distinction between “us” and “them” results in the separation of the negatively labeled group and in an increased desire for social distance from the labeled group (9). Stigmatized people often react with hurt, shame, guilt, and anger (11). Individuals who are labeled as mentally ill often develop a negative self-image due to experiences of rejection and the intrusive feeling of being different. This leads to the internalization of the stigma and the diminishment of self-esteem and self-efficacy (12). Self-stigma (13) may cause maladaptive coping strategies, such as withdrawal, and negatively impact work and social life (14). Stigmatized people face discrimination, particularly in regard to friendships, intimate relationships, education, jobs, medical treatment, and housing (2, 15).

Among people suffering from mental disorders, those with schizophrenia particularly experience negative attitudes and distancing behavior from others (4, 16, 17). During the last two decades, the desire for social distance from people with schizophrenia increased, while it remained unchanged toward people with depression or alcohol dependence (18).

The most important factor influencing stigmatization is probably the perceived danger and unpredictability associated with a particular disease by the public, because this factor is most decisive for social distance (19). People with schizophrenia are assumed to lack self-control, which makes people afraid of them and increases their desire for social distance (20). Individuals with schizophrenia and alcohol addiction are considered violent and dangerous by most people (1, 3). This fear is understandable to some degree, since those with schizophrenia have a several times higher risk of committing a violent act, with alcohol/substance abuse further increasing the risk (21–25).

### The Role of Biogenetic Explanations of Mental Disorders in Anti-stigma Campaigns

It is important to fight stigmatization, because of the devastating effects of stigmatization and discrimination on people with mental disorders. For developing successful anti-stigma campaigns, it is crucial to investigate how stigmatizing attitudes can be addressed most effectively.

Many biologically orientated researchers and several medical ethicists are optimistic that biogenetic explanations will reduce blame against persons with mental disorders, as people understand that the strange or frightening behavior is not caused by evilness or weak will, but by a disease (26–30). Many researchers hoped that the paradigm shift from a psychoanalytical and psychosocial view toward a biomedical model would have a positive influence on the stigmatization and discrimination of mental disorders (31). However, lay people still consider psychosocial factors, especially psychosocial stress, instead of biological causes, as the most common cause of mental disorders (1). Therefore, it could be expected that the introduction of biogenetic explanations (i.e., biological or genetic explanations) into anti-stigma messages might reduce the stigma associated with mental illness. Indeed, several public health programs have propagated the biogenetic model (“mental illness is an illness like any other”) (15, 32, 33). Perhaps most prominent was the slogan of the National Alliance on Mental Illness (NAMI) that mental illness is a brain disease (34).

However, many social scientists suspect that the propagation of biogenetic explanations of psychiatric disorders will intensify discrimination and stigmatization, since it will further increase feelings of fear and unfamiliarity (20, 35–38). According to this pessimistic view, “mad” or “sick” is not better than “bad,” because both attributes are negative evaluations, and the moral principles on which this relation was based remain essentially unchanged; additionally, both concepts assume an inborn predisposition for deviant behavior (39) (p. 180).

The optimistic and the pessimistic views are based on different theories of stigmatization (40). The optimistic view is based either on the attribution theory (41) or on “genetic optimism” (26). The attribution theory assumes that mainly the attribution of guilt or responsibility leads to stigmatization. If a deviant and socially undesirable behavior is caused by genetic or other biological factors, it cannot be attributed to the person because the behavior is beyond her control. Attribution theory distinguishes between onset responsibility (for getting a disease) and offset responsibility (for not being able to get well again) (12). According to the attribution theory, once the accountability assumed for a deviant behavior is reduced, social disapproval should also be reduced (41). Attribution theory predicts that stigma is reduced via reduction of blame, anger, and punishment, and increase of sympathy and helping (40). However, although explaining mental illness as a brain disease reduces blame, this approach can unintentionally exacerbate other components of stigma, especially the benevolence and dangerousness stigmas (12). Because biogenetic attributions have different effects on different aspects of stigma, it may be expected that they have different effects on different psychiatric diagnoses.

According to “genetic optimism” (26), genetic causal explanations for mental disorders make people expect effective treatments against them and, consequently, reduce the rejection of persons with mental disorders (42). Indeed, participants who received treatability information in addition to a case vignette describing a person with a mental illness attributed to a biological cause had a more positive attitude toward that person (43).

The pessimistic view is based on the observation of widespread “genetic essentialism,” i.e., “the tendency to infer a person's characteristics and behaviors as based on their perceived genetic make-up” (37) (p. 4). According to genetic essentialist thinking, genes are a person's essence, the basis of personal identity, and strongly deterministic of behavior (40, 44). In this view, mental disorders are an essential property of the person (40). In other words, the person does not have a problem; rather, the person is a problem. In this view, mental disorders belong to the core of the person or to her personal identity. Genetic essentialist thinking increases the stigma of people with mental disorders due to perceptions of differentness, persistence, seriousness, and transmissibility, which in turn increases social distance and reproductive restriction (40). Probably, the perception of dangerousness and differentness are at the core of the desire for social distance from people with mental disorders (38). Because biogenetic explanations of mental disorders might increase their perception as more dangerous and unchangeable, they might also increase the desire for social distance (1, 20, 36).

However, the attribution theory and the genetic essentialism theory are not mutually exclusive. Rather, they address different aspects of stigmatization. While the attribution theory focuses on attributing guilt, the genetic essentialism theory focuses on fear and the desire for social distance due to an inborn disposition for dangerous behavior (45). Furthermore, it is possible that geneticization has little effect on stigma, because perceptions of the behavior itself are the primary determinant of stigmatizing responses, rather than beliefs about its causes (40).

Empirical stigma research has largely confirmed the pessimistic view. Although public literacy about the biological correlates of mental disorders has increased over the last decades, attitudes toward people with mental illness did not change or worsened (33). There seems to be a “backbone of stigma” even in countries with low overall-stigma levels in spite of high levels of recognition, acceptance of neurobiological attributions, and treatment endorsement (46). Several studies found that endorsement of biogenetic explanations of mental disorders was associated with greater social distance than endorsement of psychosocial explanations (20, 36, 47–51). Some studies did not yield statistically significant associations between causal beliefs and social distance (8, 52–55). In summary, in nearly all studies, biogenetic beliefs were either associated with more desire for social distance or did not yield a statistically significant association (56). A meta-analytic review of 28 experimental studies showed that biogenetic explanations reduce blame but increase pessimism about recovery and the perception of dangerousness of people with mental disorders (57). A systematic review examining whether endorsing biogenetic causes decreases mental illness stigma in people with mental illness and in mental health professional came to a similar result as the studies with the general population (58): The majority of studies reviewed found that biogenetic causal beliefs were associated with increased stigma or negative attitudes toward mental illness (58).

However, biological causal beliefs might have an illness-specific effect: Whereas they have negative implications for depression and schizophrenia, they have some positive implications for alcohol dependence (38). Particularly for anorexia nervosa, biological explanations seem to reduce blame-based stigma (59).

These findings might explain why the campaigns propagating a biogenetic cause of mental illness failed to reduce stigmatizing attitudes. Their result was a mixed and contradictory pattern of both negative and positive emotions and cognitions, such as higher levels of negative stigma and a higher endorsement of professional treatments of mental illness (60). Since biogenetic explanations for mental diseases are associated with an increased assumption of dangerousness and unpredictability, they are not useful to reduce stigmatizing attitudes and social distance (17, 36, 42, 60).

Since previous research on stigmatization focused only on general biological explanations or specifically on genetic explanations vs. psychosocial explanations, it is not known whether different biological explanations would have different effects on stigmatizing attitudes. Few studies have compared the effects of genetic and non-genetic biological explanations on stigmatizing attitudes [e.g., (20, 38, 56, 61)].

### Aim of the Present Study

In previous papers, we hypothesized that the propagation of the mild encephalitis hypothesis of schizophrenia might reduce stigmatizing attitudes (62, 63). The present study investigates this hypothesis experimentally. To the best of our knowledge, this is the first stigmatization study to differentiate between the effects on stigmatizing attitudes of a genetic explanation and an explanation based on the mild encephalitis hypothesis.

The background of our hypothesis is the dynamic development of research on autoimmune encephalitis, mild encephalitis, and autoimmune psychosis. Autoimmune encephalitis has become an important differential diagnosis for many neuropsychiatric diseases (64, 65). A subgroup of patients diagnosed with schizophrenia has been shown to suffer from a mild chronic encephalitis caused by infection, trauma, or autoimmune diseases (66). Some patients with severe mental disorders can be successfully treated with anti-inflammatory drugs, immunosuppressive drugs, or plasma exchange (64, 67–69). Effective treatments might reduce the psychiatric symptoms and the cognitive decline enormously, prevent disablement, and allow the patients' social re-integration. The mild encephalitis hypothesis could reduce stigmatization by emphasizing the influence of infections and autoimmune diseases, which in principle can affect everyone and not only those with a specific genetic make-up (63). Furthermore, the prospect of effective treatments might reduce stigmatizing attitudes. Since the mild encephalitis hypothesis does not include genetic determinism but rather the concept of genetic vulnerability, it can be expected that the acceptance of this theory would also reduce the stigmatization of the genetic relatives of persons with schizophrenia (63). Therefore, if the mild encephalitis hypothesis proves to be medically correct and if it reduces the desire for social distance, it could be recommended to be incorporated into anti-stigma campaigns (63).

The main hypothesis of the present study is that the genetic explanation group will have the highest mean social distance score (SDS), the psychosocial explanation group the lowest mean SDS, and that the mild encephalitis hypothesis group will lie in between.

However, stigmatizing attitudes against people with mental disorders are influenced not only by the causal explanation of the disorder, but by many further factors, e.g., gender (8, 19, 51, 70–74), age (74), mental health expertise (11, 15, 16, 72, 75), education level (76, 77), and personal experience or contact with people having a mental disorder (78–81).

Therefore, we also examined the effects of several possible influencing factors and their interactions with the effect of different causal explanations on the desired social distance.

## Materials and Methods

### Participants

The study was approved by the ethics committee of the Charité - Universitätsmedizin Berlin (03.04.2020; EA4/249/19). The data were collected between April and June 2020.

A statistical power analysis was performed for sample size estimation. The effect size (ES) estimated in this study was .15, considered to be extremely small using Cohen's criteria (82). With an alpha = 0.05 and power = 0.80, the projected sample size needed with this effect size was approximately *N* = 327 questionnaires for this between comparison (GPower 3.1). Recruitment was therefore continued until this number of questionnaires had been completed.

The participants were students from different German universities who had been invited to participate in the survey. The questionnaires were distributed online via a link distributed among social networks (via Qualtrics) and in person after seminars and lectures at three universities in Berlin (paper-pencil questionnaires). The students received neither course credit nor a payment for participation. They were informed about the full voluntariness of participation, the study purpose, the full anonymity, the compliance with the EU General Data Protection Regulation, and the approval by the ethics committee of the Charité - Universitätsmedizin Berlin. The questionnaire took approximately 5–10 min to complete. Afterward, all participants could receive written information about the purpose of the experiment or ask further questions via e-mail.

### Design

The study had a prospective, quasi-experimental design that used case vignettes as independent variables (83). A questionnaire was used in three different variants. Each questionnaire consisted of one of three case vignettes, six questions for measuring the desire for social distance, and questions on the participants' gender, study discipline, and number of semesters of study. We developed three variants of a case vignette in order to assess the influence of causal explanations of schizophrenia on the desire for social distance from persons with schizophrenia. The three questionnaires (translated into English) are presented in the Supplementary Material.

Each case vignette consisted of an identical description of a person with schizophrenia, plus one of three different causal explanations for the symptoms described in the case vignette (1. genetic explanation, 2. mild encephalitis hypothesis explanation, 3. psychosocial explanation).

**Figure 1:**
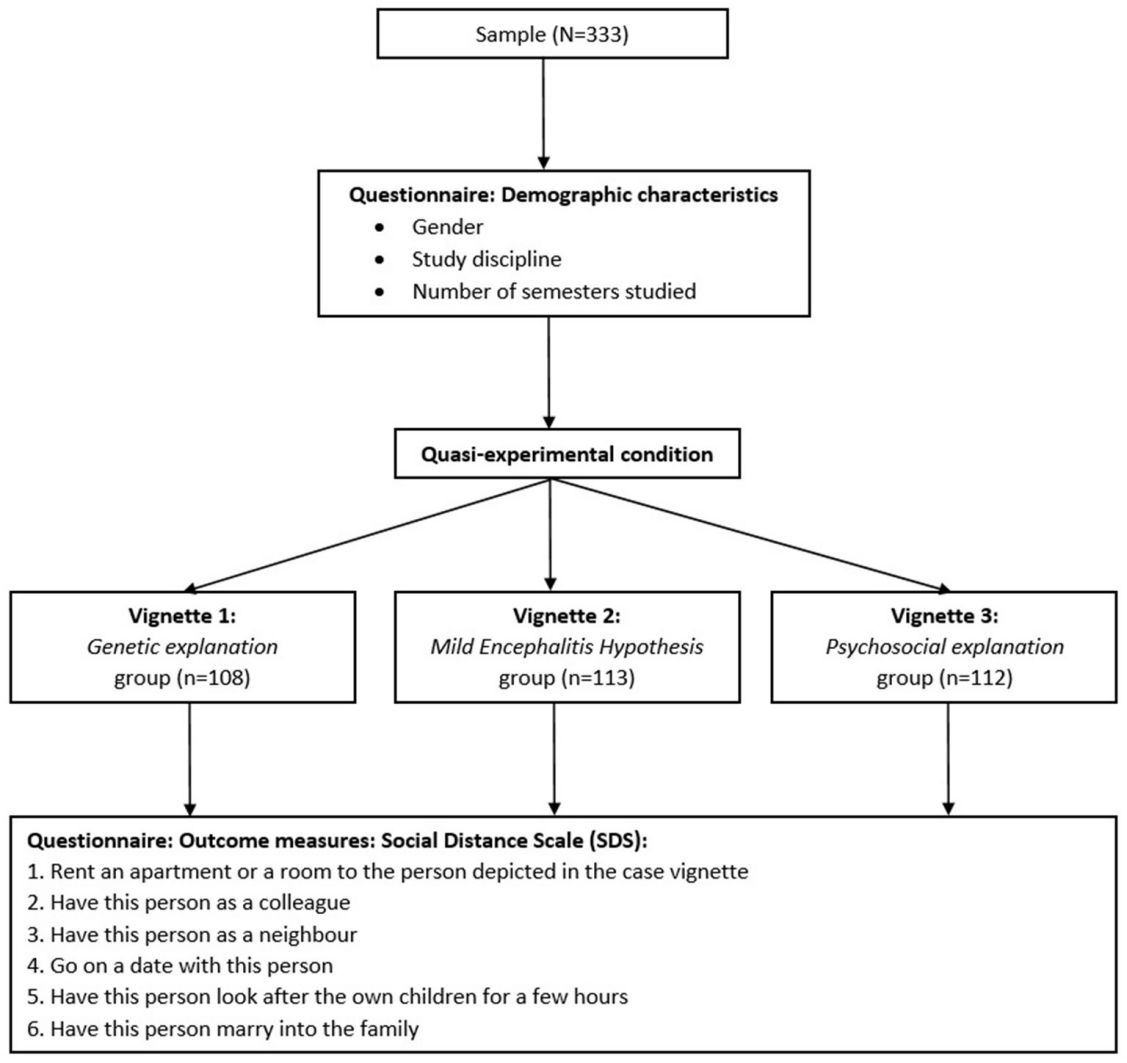
Flow chart displaying the experimental design of the vignette study and the participant flow.

The participants were randomly allocated to one of three experimental groups (1. genetic explanation, 2. mild encephalitis hypothesis explanation, 3. psychosocial explanation) (Figure 1). It was ensured that all three groups were approximately the same size.

The case vignette is based on the work of Link and Cullen (84) and Link et al. (85), who investigated the desired social distance from people with schizophrenia. It has been slightly modified and tested in a pre-study (86). The case vignette was unlabeled, i.e., the symptoms of schizophrenia were described, but not labeled as schizophrenia, so as not to activate participants' possible prior knowledge about the etiology of schizophrenia. Whereas the person in the pre-study had a male first name, the person in the present vignette was called “A.,” because in the pre-study, several participants explicitly reacted to the person's gender. For example, several male participants wrote that they would not date a man.

The general part of the case vignette and the three variants of causal explanations are presented below.

*Case vignette, general part:* Until about a year ago everything was OK with A. Then A. suddenly started to think that people were speaking derogatorily about him/her. A. was convinced that he/she was being spied on and believed that other people could read and control his/her thoughts. A. withdrew more and more, could no longer concentrate, and became increasingly apathetic, which affected his/her work. Finally, A. spent most of the time alone at home. A. heard voices saying what he/she should do and think. A. lived for more than 6 months like this until he/she was admitted to a psychiatric hospital for some time and treated with medication and psychotherapy. Now, A. is doing better, and he/she is coping well in everyday life.

1. *Variant of the case vignette: Genetic explanation:* A clinical picture such as that of A. often occurs in the course of an inheritable mental illness, for which more than 100 genes play a role.
2. *Variant of the case vignette: Mild encephalitis hypothesis:* A clinical picture such as that of A. is often caused by chronic, mild encephalitis. This is a non-lethal, mild inflammation of the brain that is chronic and causes symptoms of varying severity. The disease is caused by infection, autoimmunity, toxicity, or brain trauma.
3. *Variant of the case vignette: Psychosocial explanation:* A clinical picture such as that of A. is often due to psychosocial factors in the family environment. The mother-child relationship in early childhood plays a central role.

For measuring the desire for social distance from the person described in the case vignette, a modified version of the Social Distance Scale (SDS) of Link and Cullen (84) was used. We have modified the SDS of Link and Cullen to adapt it to the social situation of our participants, who are university students. We have replaced the question “How about having your children marry someone like Jim Johnson?” with “Would you be OK with A. marrying into your family?”. Furthermore, we have added a question with special importance for young adults: “Would you go on a date with A.?”. The modified version of the SDS has also been tested in the pre-study (86).

The adapted social distance scale (SDS) includes six items representing different social relationships: (1). rent an apartment/room to the person depicted in the case vignette, (2). have this person as a colleague, (3). have this person as a neighbor, (4). go on a date with this person, (5). have this person as the caretaker of one's children for a couple of hours, (6). have this person marry into the family. The responses were assessed on a 5-point Likert Scale, indicating to what extent the participants would be willing or unwilling to engage in a certain relationship (0 = *no answer*, 1 = *definitely yes*, 2 = *rather yes*, 3 = *rather no*, 4 = *definitely no*). For the total mean SDS score, the scores for each item per group were added and divided by 6. Internal consistency (Cronbach's α) was measured as .79. Values ranged from 1 to 4, with a higher value indicating a stronger desired social distance.

### Statistical Analysis

For the analysis, IBM® SPSS Statistics® (version 25) was used.

The independent variable was the causal explanation consisting of three variants (1. genetic, 2. mild encephalitis hypothesis, 3. psychosocial). The dependent variable was the total mean SDS score. To explore the association between the causal explanations and the total mean SDS score, a one-way analysis of variance (ANOVA) was conducted.

Three subgroup analyses were performed to assess main and potential interaction effects of gender, study discipline, and number of semesters, with a two-way ANOVA in each case. For the analysis, the study discipline was categorized into three different groups: (1) social sciences and economics, (2) scientific and technical engineering mathematics (STEM), and (3) health care and pedagogics (including psychology, medicine, and pedagogics). Number of semesters of study were categorized into two groups: (1) 1–6 semesters, and (2) 7+ semesters.

As the questions in the SDS focus on different degrees of relationship with the person described, they were analyzed individually with the Kruskal-Wallis test as the non-parametric equivalent of the one-way ANOVA.

## Results

In total, 333 evaluable questionnaires were collected. The genetic explanation was received by 108 participants (32.43%), the mild encephalitis hypothesis explanation by 113 (33.93%), and the psychosocial explanation by 112 (33.63%). Of the participants, 124 (37.24%) were male, 209 (62.76%) female, and 0 (0%) diverse. 152 (45.6%) participants studied social sciences and economics, 78 (23.42%) STEM, and 103 (30.93%) health care and pedagogics. 152 (45.65%) participants studied between 1–6 semesters, and 181 (54.35%) 7+ semesters. The average number of completed semesters was 6.98. Table 1 shows the comparison of baseline characteristics among the groups.

**Table 1:** Comparison of baseline characteristics among the groups.

	**Genetic explanation**	**Mild encephalitis hypothesis explanation**	**Psychosocial explanation**	**Row sum**
**Gender**				
Female	66	71	72	209
Male	42	42	40	124
Diverse	0	0	0	0
*Total*	108	113	112	333
**Study discipline**				
STEM	24	27	27	78
Social sciences and economics	55	47	50	152
Health care and pedagogics	29	39	35	103
*Total*	108	113	112	333
**Number of semesters**				
1–6 semesters	53	51	48	152
7+ semesters	55	62	64	181
*Total*	108	113	112	333

### Main Analysis

**Figure 2:**
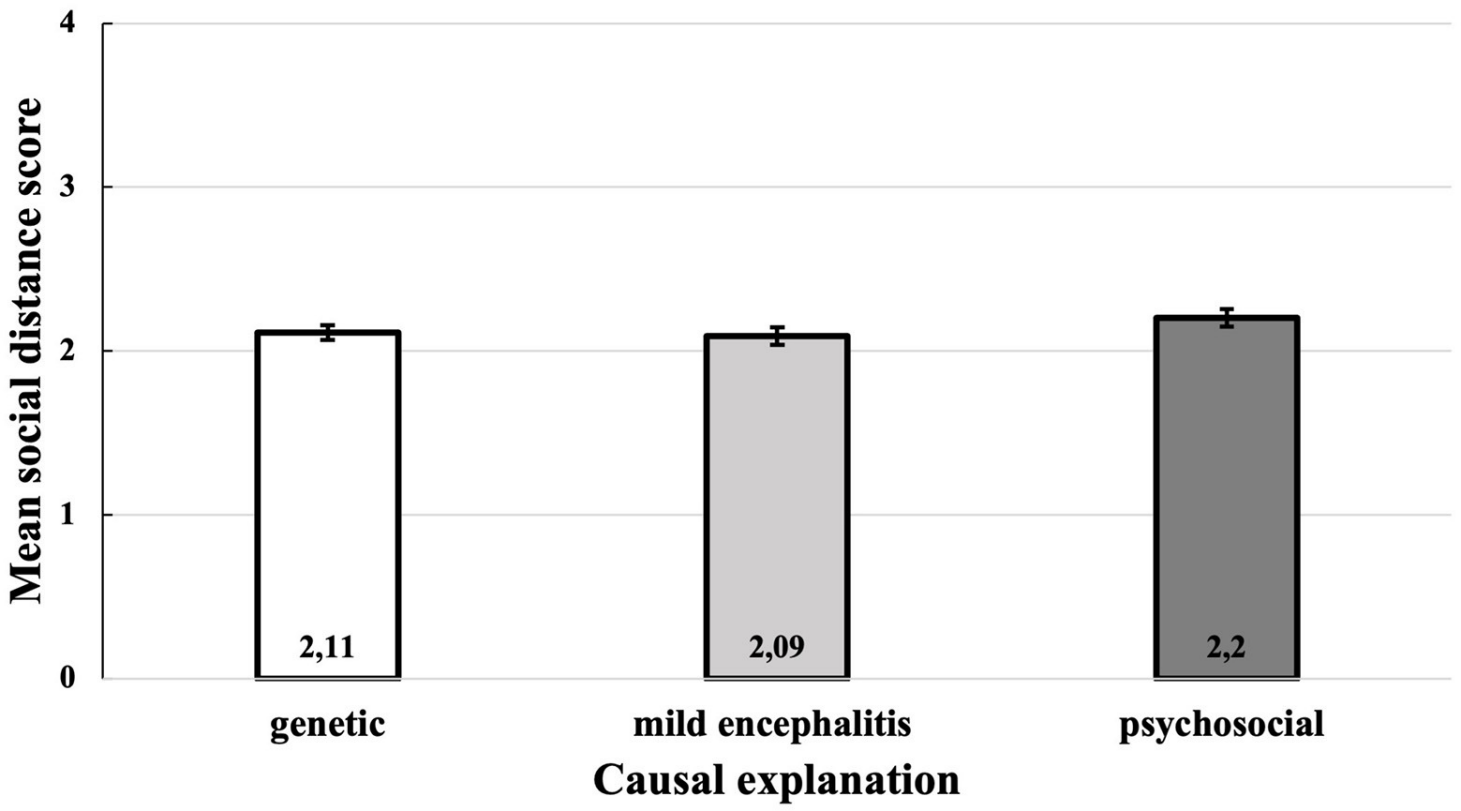
Mean social distance score as a function of the causal explanation.

To examine the effect of causal explanations of schizophrenia on the total mean SDS score, a one-way between subjects ANOVA was conducted. The descriptive statistics in Table 2 show that participants who received the psychosocial causal explanation had the highest mean SDS score, followed by the group with the genetic explanation. The group with the mild encephalitis hypothesis had the lowest mean SDS score (see Figure 2). However, these differences were small and there is no significant effect of the causal explanation of schizophrenia on the desired social distance at the *p* < 0.05 level, *F*_(2, 330)_ = 1.37, *p* = 0.257.

**Table 2:** Descriptive statistics for the total mean social distance score as function of the causal explanation.

**Causal explanation**	***N***	***M***	***SD***	***95% CI***	
genetic	108	2.110	0.467	2.021	2.199
mild encephalitis hypothesis	113	2.091	0.553	2.094	2.306
psychosocial	112	2.200	0.566	1.988	2.194

*N, number of participants; M, Mean SDS score; SD, standard deviation; 95% CI, 95% confidence interval*.

### Subgroup Analyses

#### Gender

The descriptive statistics of gender are presented in Table 3.

**Table 3:** Descriptive statistics for the total mean social distance score as function of gender.

**Gender**	***N***	***M***	***SD***	***95% CI***	
Male	124	2.222	0.527	2.128	2.313
Female	209	2.081	0.529	2.008	2.150
Diverse	0	-	-	-	-
*Total*	333	2.134	0.532	2.076	2.191

*N, number of participants; M, Mean SDS score; SD, standard deviation; 95% CI, 95% confidence interval*.

A two factor ANOVA was conducted to evaluate the effect of the causal explanations and gender on the total mean SDS score. As no participant selected “gender: diverse,” only two genders were considered, resulting in a 2 × 3 instead of a 3 × 3 matrix. The two independent variables in this analysis were gender (male, female) and causal explanation (genetic, mild encephalitis hypothesis, psychosocial). The results are presented in Supplementary Table 1.

**Figure 3:**
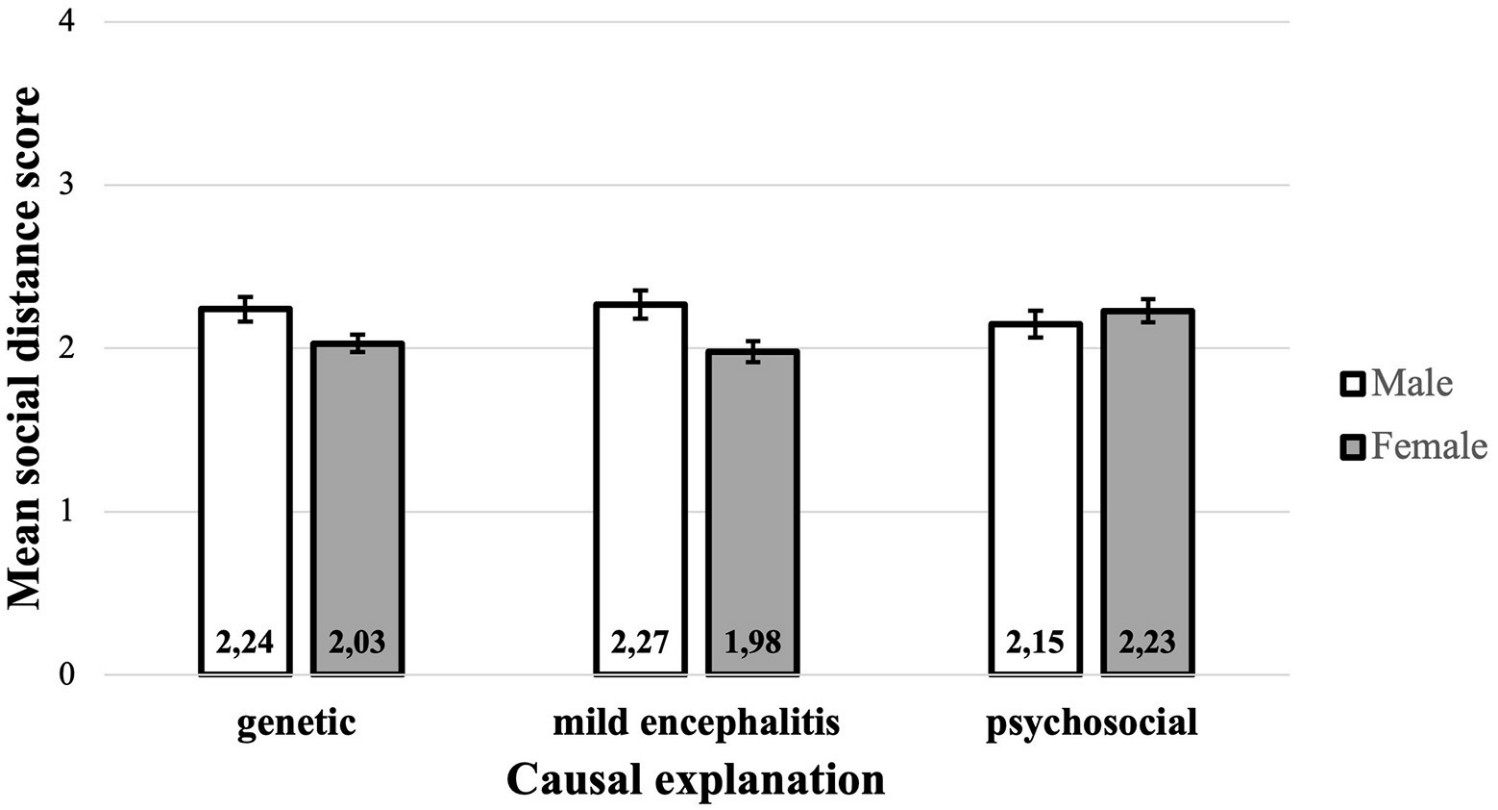
Interaction between gender and causal explanation.

A significant main effect for gender was found (*p* = 0.018), indicating that women have lower social distance scores (*M* = 2.081, *SD* = 0.529, 95% *CI* [2.008, 2.150]) than men (*M* = 2.222, *SD* = 0.527, 95% *CI* [2.128, 2.313]). No main effect for the causal explanation was identified (*p* = 0.653). There was a significant interaction between gender and causal explanation (*p* = 0.026), which indicates that any difference between the total mean SDS score depends on gender, and that any difference between males and females depends on the causal explanation. Since the interaction between gender and causal explanation was significant, the main effect was ignored and the gender simple main effects, which are the differences between males and females for each of the three causal explanations, were examined. To control for Type I error across the three simple main effects, the alpha level was set at.017 (α/3 = 0.05/3). After a pairwise comparison, the only significant difference between males and females was found in the group that received the mild encephalitis hypothesis as a causal explanation. In the mild encephalitis group, the total mean SDS score of men (*M* = 2.28, *SD* = 0.53, 95% *CI* [2.116, 2.434]) was significantly higher than that of women (*M* = 1.98, *SD* = 0.51, 95% *CI* [1.859, 2.104]) (*p* = 0.04). Even though the included predictors had some predictive value, the variance explained by the model was rather small (adjusted R^2^ = 0.032). For the genetic causal explanation, a similar trend can be observed, with men showing a higher SDS score than women. In contrast, in the psychosocial explanation group, the mean SDS score of men was smaller than that of women (Figure 3). However, the results in the groups that received either the genetic or the psychosocial explanation showed no significant gender differences within the groups.

#### Study Discipline

Table 4 shows the descriptive statistics of the study discipline. The results show a tendency for health care and pedagogics students to have the lowest total mean SDS score, whereas the STEM students have the highest total mean SDS score. However, the difference is not statistically significant (*p* = 0.064).

**Table 4:** Descriptive statistics for social distance score as function of study discipline.

**Study discipline**	***N***	***M***	***SD***	**95%** ***CI***	
Social science and economics	152	2.117	0.556	2.028	2.207
STEM	78	2.250	0.509	2.135	2.365
Health care and pedagogics	103	2.069	0.501	1.972	2.167

*N, number of participants; M, Mean SDS score; SD, standard deviation; 95% CI, 95% confidence interval*.

#### Number of Semesters of Study

The descriptive statistics for the number of semesters of study is presented in Table 5. Participants who studied between 1–6 semesters have a higher SDS score than those who studied 7+ semesters, but the difference was not statistically significant (*p* = 0.075).

**Table 5:** Descriptive statistics for social distance score as function of number of semesters.

**Number of semesters**	**N**	***M***	***SD***	**95%** ***CI***	
1–6	152	2.188	0.512	2.106	2.270
7+	181	2.088	0.545	2.008	2.168

*N, number of participants; M, Mean SDS score; SD, standard deviation; 95% CI, 95% confidence interval*.

#### Individual Questions of the SDS

**Figure 4:**
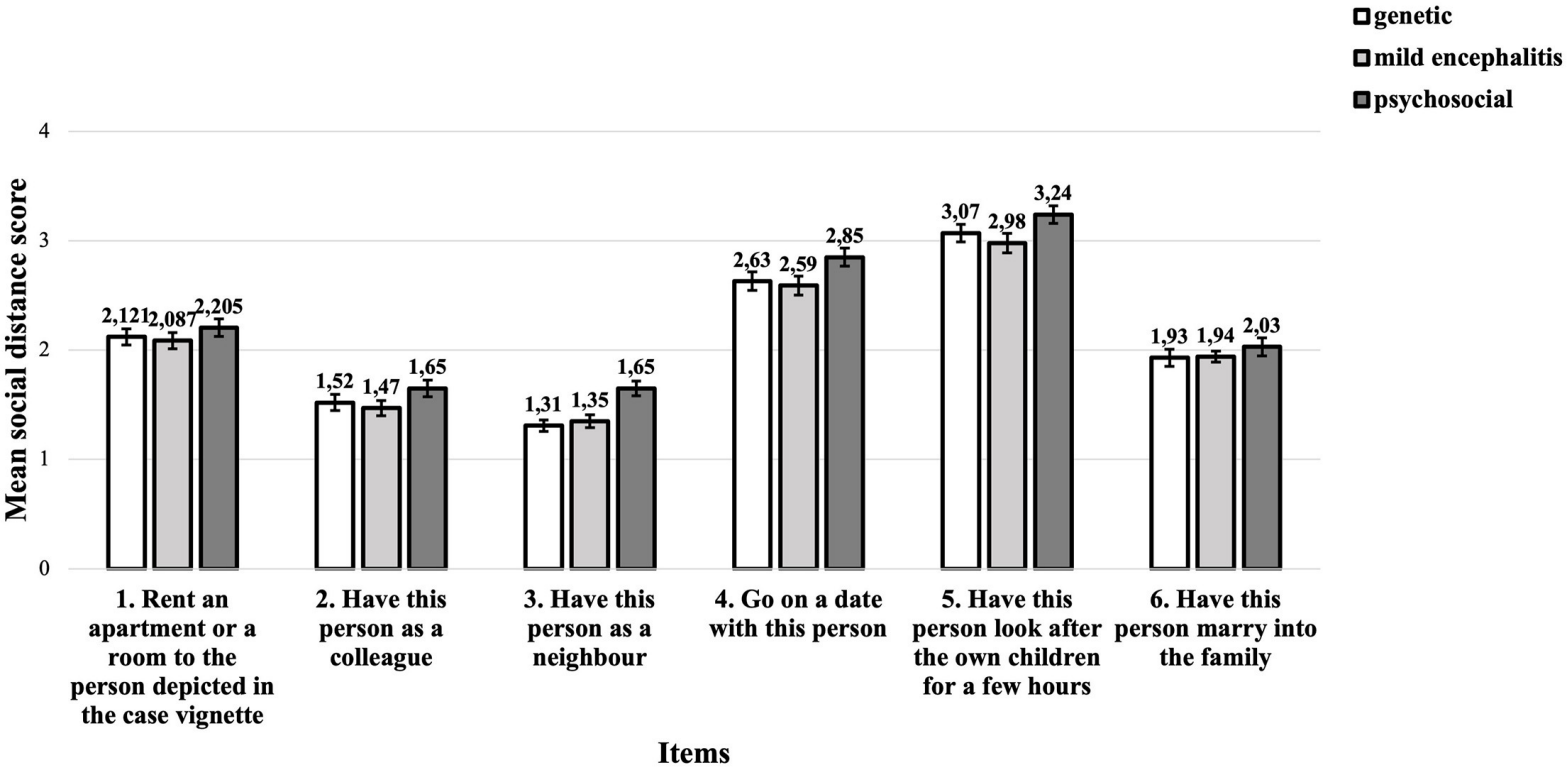
Mean social distance score as a function of items and given causal explanation.

Table 6 shows the mean social distance scores for each question. Figure 4 shows the mean social distance scores for each question and for each causal explanation. The scores of the individual questions were examined for significant differences between the groups (see Supplementary Table 2). None of the individual questions showed a significant difference between the groups.

**Table 6:** Mean social distance score for the six questions.

**Item**	**1**	**2**	**3**	**4**	**5**	**6**
*M*	2.140	1.560	1.380	2.720	3.090	1.950
*SD*	0.076	0.706	0.588	0.806	0.806	0.822

*M, Mean SDS score; SD, standard deviation*.

The differences between the mean SDS scores for the different items are much bigger than the differences for the different causal explanations. The biggest concerns are about having a person with schizophrenia look after the own children for a few hours, followed by concerns about going on a date with this person. The smallest concerns are about having this person as a neighbor. However, the different causal explanations hardly influence these concerns.

## Discussion

The main hypothesis of this study was that the genetic explanation has the strongest stigmatizing effect, and the psychosocial explanation the weakest, and that the mild encephalitis hypothesis explanation lies between them. The results of the present study did not find statistically significant different effects of different causal explanations of schizophrenia on the desired social distance from people suffering from schizophrenia. However, the sample showed the tendency that participants who received a biogenetic causal explanation expressed a lesser desire for social distance compared to participants who received a psychosocial causal explanation.

The few studies that have compared the effects of genetic and non-genetic biological explanations of mental disorders on stigmatizing attitudes came to inconsistent results. Dietrich et al. found that the more respondents endorsed a “brain disease” as a cause of schizophrenia, the more dangerous they believed the person with schizophrenia to be (20). The relationship between the endorsement of “heredity” and perceived dangerousness was less pronounced than for “brain disease” (20). By contrast, Rüsch et al. found that the endorsement of a genetic explanation was associated with increased social distance, whereas the endorsement of a “brain disorder caused by biological changes in brain metabolism” was not significantly associated with increased social distance (61) (p. 329). Angermeyer et al. found a statistically significant association between the endorsement of brain disease but not for the endorsement of hereditary factors as a cause of schizophrenia and social distance (56). They found that the endorsement of both “chemical imbalance of the brain” and “brain disease” as cause of schizophrenia was associated with an increased social distance, whereas “heredity” was not significantly associated with social distance (38). A meta-analytic review found that general biogenetic explanations and neurochemical explanations, but not genetic explanations, were associated with stigmatizing attitudes (87).

The mild encephalitis hypothesis might be promising in tackling public stigma, because it would enable medical treatment options (63). If people were informed about possible treatment options for schizophrenia based on the mild encephalitis hypothesis, this might reduce stigmatizing attitudes. This assumption is based on the results of the previously mentioned study of Lebowitz and Ahn, which showed that participants who received treatability information, in addition to a case vignette describing a person with a mental illness attributed to a biological cause, had a more positive attitude toward that person (43). However, in the present study, the participants received only causal explanations of the symptoms, but no information about treatment options, suggesting that many participants might not have actively considered them. Therefore, it is possible that the additional information about treatment options based on the mild encephalitis hypothesis would have resulted in a lower SDS score.

An unexpected finding of the present study was the interaction between gender and the received causal explanation on the desired social distance. Female participants showed a significantly smaller desired social distance than male participants when provided with the mild encephalitis hypothesis. Furthermore, a trend could be observed that women who received the genetic causal explanation showed less desire for social distance compared to men, whereas men in the group that received the psychosocial causal explanation showed a lower tendency for social distance. However, these differences are very small and not significant.

The influence of gender on stigmatizing attitudes has been reported inconsistently. Some studies found higher stigmatizing attitudes and desire for social distance in women (51, 70), other studies in men (19, 71–74). Some studies did not find significant differences (8, 88). Angermeyer et al. (88) argued that, on the one hand, women have more pro-social attitudes, but on the other hand, women experience greater fear than men do, so that these effects might neutralize each other. A study with medical students supports this hypothesis (80). Therefore, the results of the present study must be interpreted with caution. Further research is necessary to investigate the complex relationship between gender and causal explanations on stigmatization.

The present study shows a non-significant trend that health care and pedagogics students had a lower SDS than other students. However, stigmatizing attitudes are not uncommon among health professionals (89) and mental health professionals (11, 90), as well as general practitioners and medical students (79). However, there is contradictory evidence for the influence of mental health expertise on stigmatizing attitudes (15, 16, 75).

The present study found a non-significant trend that students who studied 7+ semesters had a lower SDS score than students with fewer semesters did. This result is consistent with several surveys of the general population, which have consistently shown that higher education is linked to lesser desired social distance from people with mental disorders (76, 77).

Lastly, each question of the SDS was assessed individually because the items presented social relationships of varying intensity. We found that, regardless of the causal explanation, the extent of the desired social distance depends strongly on social proximity. The participants particularly refuse to have the person with schizophrenia symptoms look after their children and to go on a date with this person. This result confirms previous research, which has shown that the level of social distance is higher the greater the level of social closeness is (51, 91). It is in line with Pescosolido et al.'s finding that “issues that deal primarily settings (the family), vulnerable groups (children), or self-harm elicit the greatest amount of negative response” (46) (p. e5). Furthermore, this result supports the assumption that social distance is strongly based on fear. The most decisive factor for social distance is probably the perceived danger and unpredictability associated with a particular disease (19). Especially dating a stranger and entrusting one's own child to a stranger can trigger fear for good reasons, especially in women.

### Recommendations

Like many previous studies, the present study supports the hypothesis that factors other than the causal explanation for schizophrenia significantly influence social distance, suggesting that providing people with information about the etiology of schizophrenia will not reduce the stigmatization. Therefore, anti-stigma campaigns should not focus only on the causes of the disease. Rather, they should provide information on effective treatments based on causal explanations of schizophrenia. If people learn that effective treatments are available which can reduce the frightening symptoms, this information might effectively reduce their fear and their desire for social distance. Therefore, we recommend incorporating information on treatment options based on the mild encephalitis hypothesis into anti-stigma campaigns.

Although this study found significantly less social distance toward persons with schizophrenia in women who received the mild encephalitis hypothesis, we do not recommend gender-specific anti-stigma campaigns. This is because, first, the anti-stigmatizing effect is small. Secondly, there are no media that are not used across genders. Thirdly, target group-specific campaigns give the impression that they are intended to manipulate and deceive.

We support the recommendation for use of recovery-oriented messages and “see the person” messages, which have been developed by 32 experts attending an international conference on mental health stigma (92). In this regard, the mild encephalitis hypothesis could contribute to recovery-oriented messages if examples can be used to show how patients diagnosed with schizophrenia can be cured by anti-inflammatory and immunomodulatory therapies.

## Limitations

The present study has several limitations. First, the data of the present study are based on a convenience sample consisting of German university students. Therefore, the results are not representative for the general German population. Moreover, the results are likely to be transferable to other Western industrialized countries (33), but presumably not to different cultural contexts. Second, the SDS, although modeled after the SDS of Link and Cullen (84), was developed exclusively for the present study without having been validated elsewhere. Third, although several influence factors on stigmatizing attitudes have been considered, the factor of personal familiarity with schizophrenia or contact with people having schizophrenia has not been considered. Fourth, as in previous similar studies, no control group that did not receive any causal explanation for the symptoms was used. Fifth, despite the complete and secure anonymity, the answers are likely influenced by social desirability concerns.

## Data Availability Statement

The raw data supporting the conclusions of this article will be made available by the authors, without undue reservation.

## Ethics Statement

The studies involving human participants were reviewed and approved by Ethics committee of the Charité - Universitätsmedizin Berlin, CCM (03.04.2020; EA4/249/19). The participants provided their written informed consent to participate in this study.

## Author Contributions

SM: development of the concept and revision of the paper. SH and SM: writing of the paper and figures and tables. SH, SM, and CH: literature research. SH and CH: statistical analysis. All authors contributed to the article and approved the submitted version.

## Conflict of Interest

The authors declare that the research was conducted in the absence of any commercial or financial relationships that could be construed as a potential conflict of interest.

## Publisher's Note

All claims expressed in this article are solely those of the authors and do not necessarily represent those of their affiliated organizations, or those of the publisher, the editors and the reviewers. Any product that may be evaluated in this article, or claim that may be made by its manufacturer, is not guaranteed or endorsed by the publisher.

## Abbreviations

SDS: Social Distance Scale
NAMI: National Alliance on Mental Illness.
