# Supplementary material for: Comparison of the Effects of a Genetic, a Mild Encephalitis, and a Psychosocial Causal Explanation of Schizophrenia on Stigmatizing Attitudes – a Pilot Study With a Quasi-Experimental Design
Source: Front Psychiatry. 2021 Sep 20;12:745124. doi: 10.3389/fpsyt.2021.745124 (PMC8489806; doi:10.3389/fpsyt.2021.745124)
Supplement: Supplementary file 1 [file Data_Sheet_1.pdf]

Supplementary Material

Sonja Haouchet, Carolin Harder and Sabine Müller

Comparison of the effects of a genetic, a mild encephalitis, and a psychosocial causal explanation of schizophrenia on stigmatizing attitudes – a pilot study with a quasi-experimental design

Questionnaires

1. Questionnaire: Genetic explanation

2. Questionnaire: Mild encephalitis hypothesis explanation

3. Questionnaire: Psychosocial explanation

Tables

S-1: Results of the 2*3 ANOVA for evaluating the effect of the causal explanations and gender on the total mean SDS score

S-2: Kruskal-Wallis Test for independent samples with the group variable SDS

CHARITÉ

CharitéCentrum for Neurology, Neurosurgery and Psychiatry

Department of Psychiatry and Neurosciences, CCM

Study information: My name is Sonja Haouchet and I study neuropsychology at Maastricht University in the Netherlands. As part of my master's thesis under the supervision of PD Dr. Sabine Müller from the Department of Psychiatry and Neurosciences, CCM, at Charité - Universitätsmedizin Berlin, I am conducting a study on the stigmatization of mentally ill people.

Privacy information: Your complete anonymity is guaranteed. All responses will be reported only in aggregate form. Data protection in accordance with the General Data Protection Regulation (GDPR) is guaranteed. By submitting the completed questionnaire, you consent to participate in the study.

Please read the following case vignette and answer the following questions.

Case vignette: Until about a year ago everything was okay with A. Then A. suddenly started to think that people were talking badly and derogatorily about him/her. A. was convinced that he/she was being spied on and believed that other people could read and control his/her thoughts. A. withdrew more and more, could no longer concentrate well and became increasingly apathetic, which also affected his/her work. Finally, A. spent most of the time alone at home. A. heard voices saying what he/she should do and think. Therefore, A. lived for more than 6 months like this until he/she was admitted to a psychiatric hospital for some time and treated with medication and psychotherapy. In the meantime, A. is doing better, and he/she is coping well in everyday life.
Information about the case: A clinical picture such as that of A. often occurs in the course of an inheritable mental illness, for which more than 100 genes play a role.

Questions:

1. Would you rent/sublet your room/apartment to A.?
O definitively no O rather no O rather yes O definitively yes O no answer

2. Would you be okay with having A. as a colleague?
O definitively no O rather no O rather yes O definitively yes O no answer

3. Would you be okay with having A. as a neighbor?
O definitively no O rather no O rather yes O definitively yes O no answer

4. Would you go on a date with A.?
O definitively no O rather no O rather yes O definitively yes O no answer

5. Would you have A. as the caretaker of your children for a couple of hours?
O definitively no O rather no O rather yes O definitively yes O no answer

6. Would you be okay with A. marrying into your family?
O definitively no O rather no O rather yes O definitively yes O no answer

Gender (M/W/D): ……. Study discipline: …………………………………. Number of semesters: ……

Thank you very much for your participation!
For further inquiries you reach me at: xxx.

CHARITÉ

CharitéCentrum for Neurology, Neurosurgery and Psychiatry

Department of Psychiatry and Neurosciences, CCM

Study information: My name is Sonja Haouchet and I study neuropsychology at Maastricht University in the Netherlands. As part of my master's thesis under the supervision of PD Dr. Sabine Müller from the Department of Psychiatry and Neurosciences, CCM, at Charité - Universitätsmedizin Berlin, I am conducting a study on the stigmatization of mentally ill people.

Privacy information: Your complete anonymity is guaranteed. All responses will be reported only in aggregate form. Data protection in accordance with the General Data Protection Regulation (GDPR) is guaranteed. By submitting the completed questionnaire, you consent to participate in the study.

Please read the following case vignette and answer the following questions.

Case vignette: Until about a year ago everything was okay with A. Then A. suddenly started to think that people were talking badly and derogatorily about him/her. A. was convinced that he/she was being spied on and believed that other people could read and control his/her thoughts. A. withdrew more and more, could no longer concentrate well and became increasingly apathetic, which also affected his/her work. Finally, A. spent most of the time alone at home. A. heard voices saying what he/she should do and think. Therefore, A. lived for more than 6 months like this until he/she was admitted to a psychiatric hospital for some time and treated with medication and psychotherapy. In the meantime, A. is doing better, and he/she is coping well in everyday life.
Information about the case: A clinical picture such as that of A. is often caused by chronic, mild encephalitis. This is a non-letal, mild inflammation of the brain that is chronic and causes symptoms of varying severity. The disease is caused by infection, autoimmunity, toxicity or brain trauma.

Questions:

1. Would you rent/sublet your room/apartment to A.?
O definitively no O rather no O rather yes O definitively yes O no answer

2. Would you be okay with having A. as a colleague?
O definitively no O rather no O rather yes O definitively yes O no answer

3. Would you be okay with having A. as a neighbor?
O definitively no O rather no O rather yes O definitively yes O no answer

4. Would you go on a date with A.?
O definitively no O rather no O rather yes O definitively yes O no answer

5. Would you have A. as the caretaker of your children for a couple of hours?
O definitively no O rather no O rather yes O definitively yes O no answer

6. Would you be okay with A. marrying into your family?
O definitively no O rather no O rather yes O definitively yes O no answer

Gender (M/W/D): ……. Study discipline: …………………………………. Number of semesters: ……

Thank you very much for your participation!
For further inquiries you reach me at: xxx.

CharitéCentrum for Neurology, Neurosurgery and Psychiatry

Department of Psychiatry and Neurosciences, CCM

Study information: My name is Sonja Haouchet and I study neuropsychology at Maastricht University in the Netherlands. As part of my master's thesis under the supervision of PD Dr. Sabine Müller from the Department of Psychiatry and Neurosciences, CCM, at Charité - Universitätsmedizin Berlin, I am conducting a study on the stigmatization of mentally ill people.

Privacy information: Your complete anonymity is guaranteed. All responses will be reported only in aggregate form. Data protection in accordance with the General Data Protection Regulation (GDPR) is guaranteed. By submitting the completed questionnaire, you consent to participate in the study.

Please read the following case vignette and answer the following questions.

Case vignette: Until about a year ago everything was okay with A. Then A. suddenly started to think that people were talking badly and derogatorily about him/her. A. was convinced that he/she was being spied on and believed that other people could read and control his/her thoughts. A. withdrew more and more, could no longer concentrate well and became increasingly apathetic, which also affected his/her work. Finally, A. spent most of the time alone at home. A. heard voices saying what he/she should do and think. Therefore, A. lived for more than 6 months like this until he/she was admitted to a psychiatric hospital for some time and treated with medication and psychotherapy. In the meantime, A. is doing better, and he/she is coping well in everyday life.
Information about the case: A clinical picture such as that of A. is often due to psychosocial factors in the family environment. Especially the mother-child relationship in early childhood plays a central role.

Questions:

1. Would you rent/sublet your room/apartment to A.?
O definitively no O rather no O rather yes O definitively yes O no answer

2. Would you be okay with having A. as a colleague?
O definitively no O rather no O rather yes O definitively yes O no answer

3. Would you be okay with having A. as a neighbor?
O definitively no O rather no O rather yes O definitively yes O no answer

4. Would you go on a date with A.?
O definitively no O rather no O rather yes O definitively yes O no answer

5. Would you have A. as the caretaker of your children for a couple of hours?
O definitively no O rather no O rather yes O definitively yes O no answer

6. Would you be okay with A. marrying into your family?
O definitively no O rather no O rather yes O definitively yes O no answer

Gender (M/W/D): ……. Study discipline: …………………………………. Number of semesters: ……

Thank you very much for your participation!
For further inquiries you reach me at: xxx.

Table S-1: Results of the 2*3 ANOVA for evaluating the effect of the causal explanations and gender on the total mean SDS score

Source	Sum of Squares	*df*	Mean Square	*F*	Sig.	Partial Eta Squared
Corrected Model	4.368[a]	5	0.874	3.193	0.008	0.047
Intercept	1435.801	1	1437.801	5254.533	0.000	0.941
Causal Explanation	0.234	2	0.117	0.427	0.653	0.003
Gender	1.560	1	1.560	5.703	0.018	0.017
Causal Explanation * Gender	2.010	2	1.005	3.672	0.026	0.022
Error	89.477	327	0.274			
Total	1609.792	333				
Corrected Total	93.846	332				

a. R Squared = .046 (Adjusted R Squared = .032)

df = degrees of freedom. *F* = Fisher's F Ratio. Sig. = significance

Table S-2: ***Kruskal-Wallis Test for independent samples with the group variable SDS***

**Item**	**1**	**2**	**3**	**4**	**5**	**6**
Kruskal-Wallis H	3.392	2.891	0.489	2.423	4.161	0.802
df	2	2	2	2	2	2
Sig.	0.183	0.236	0.783	0.298	0.125	0.670

df = degrees of freedom. Sig = significance. p-value of 0.05 is considered statistically significant
